# Multiple Authors in a Scientific Article: Friends and Foes

**DOI:** 10.1093/icvts/ivag224

**Published:** 2026-08-17

**Authors:** Carlos A Mestres, Andres Obeso, Laszlo Göbölös

**Affiliations:** Department of Cardiothoracic Surgery and The Robert WM Frater Cardiovascular Research Centre, The University of the Free State, Bloemfontein, 9301, South Africa; Heart, Vascular & Thoracic Institute, Cleveland Clinic Abu Dhabi, Abu Dhabi, United Arab Emirates; Heart, Vascular & Thoracic Institute, Cleveland Clinic Abu Dhabi, Abu Dhabi, United Arab Emirates

According to sources, “academic(s)” represent activities related to schools, colleges, and universities, or connected with studying and thinking, not with practical skills; and “career,” the job or series of jobs that someone does during a working life, especially if one continues to get better jobs and earn more money. Furthermore, “knowledge” is the understanding of, or information about a subject that one gets by experience or study, either known by one person or by people generally.^1^

Having said that, it is known that the medical community dedicates a substantial proportion of time to gaining knowledge for the benefit of specific patients and society. Direct interaction and patient care, education, and research are the 3 basic pillars of healthcare. Currently, especially at the tertiary or quaternary hospital/university level, basic and clinical research are part of the professional life of all involved in patient care, from physicians to nursing staff and biomedical engineering, to say the least. Thus, transferring knowledge acquired in the laboratory or in the clinic and hospital is seen as a fundamental component of the medical profession.

Scientific publications, then, currently have a strong impact on professional careers. Departments and institutions, above all, universities, are keen on “collecting” publications aiming at, among other objectives, increasing institutional prestige that may result in climbing the positions in ranking tables, greater attraction of the so-called healthcare customers—formerly patients—and overall improving key performance indicators and financial balance. What makes things more complicated is that medical knowledge is exponentially growing, and more and more efforts of all kinds are invested in scientific publishing. According to a decade-old illuminating and enlightening contribution by Densen, the estimated doubling time of medical knowledge in 1950 was 50 years; in 1980, 7 years; in 2010, 3.5 years. The projection for the 2020 decade was 0.2 years, or just 73 days.^2^ This is 2026, today.

This means that knowledge is no longer in the hands of given isolated individuals and that multidisciplinary work is the way to go. The concept of “publish or perish,” which entails competition at its fiercest, has rapidly been introduced in academic life, to describe the pressure to publish academic work to succeed in an academic career as defined above. This raises issues related to integrity and objectivity,^3^ among other issues. As a consequence of multidisciplinary work, a mounting number of researchers are involved in studies.

In this issue of the Journal, Massey et al^4^ discuss multiple authorship in the cardiothoracic surgical literature, in fact, the continuation of their previous work on the topic almost 2 decades ago.^5^ This is a simple but interesting bibliometric analysis with a focus on the 3 leading cardiothoracic journals (European Journal of Cardiothoracic Surgery, EJCTS; The Journal of Thoracic and Cardiovascular Surgery, JTCVS; and The Annals of Thoracic Surgery, ATS). After digging deep into more than 17,000 published articles, the authors have clearly confirmed that the mean number of authors per article increased over the past 2 decades for all journals. Of interest is to notice that internationalization of authorship has mostly and only been observed in the EJCTS.

Additional remarkable figures include the fact that single/double authorship is disappearing and that articles with 6 or more authors represent the largest proportion in academic output. Massey et al^4^ discuss possible factors that contribute to the increase in multiple authorship and draw the attention of the readership to burning issues like major ethical concerns, as the actual role of all authors in a given article may be a matter of discussion.

Notwithstanding the raw data produced by the authors and the trends observed over 2 decades in the 3 major cardiothoracic journals, the contribution of Massey et al^4^ has not explored consensus documents or basic research articles which, mostly out of the reach of the JTCVS, ATS, and EJCTS, may gather an authorship of over 100 or even 200 authors. This was not the primary objective of the authors, but it would have been of interest to the readership. Some other aspects that could have been explored are how the decision on the authors’ origin was made, by name, primary employment, matching date of publication with the given employment at that time…? It is also somewhat surprising, despite the affiliation of the authors, that the United Kingdom was not included in the European pool of articles, rather than being separated, considering the 20-year study window. Furthermore, the issue of ghostwriters would further skew the analytic balance. Another problem in current research, namely the major ethical issue of retraction of scientific articles, has not been a part of the authors’ objectives, although we know that breach of ethics is always beneath retraction. In any case, the community can address retractions through a specific database, a blog reporting on retractions of scientific papers and on related topics.^6^

With all the possible weaknesses of the study by Massey et al,^4^ they insist on a critical aspect of research, the credibility and quality of scientific research through transparency. Back to “publish or perish,” this cultural paradigm entails risks of bias, suboptimal academic work, or even fraud. Perhaps some other ways to foster an academic career should be contemplated, like the very recent Research Integrity Risk Index (RI²) which aims at assessing institutional exposure to selected research-integrity-related risks like retracted journal articles, and institutional self-citation patterns.^7^ Or simply, assuming that career planning might need subspecialty-specific analysis considering the differences across specialties^8^ and, as rightly stated by Massey et al,^4^ that all individuals listed as authors meet the criteria for meaningful contributions.

## Data Availability

Data are publicly available as all this information has been extracted after careful review of the literature.

## References

[1] https://dictionary.cambridge.org/dictionary/english.

[2] Densen P. Challenges and opportunities facing medical education. Trans Am Clin Climatol Assoc. 2011;122:48-58. 21686208 PMC3116346

[3] Fanelli D. Do pressures to publish increase scientists’ bias? An empirical support from US states data. PLoS One. 2010;5:e10271. 20422014 10.1371/journal.pone.0010271 PMC2858206

[4] Massey J , Zahid Z, Shaw M, Modi P. Continuing authorship proliferation in the cardiothoracic surgical literature. Interdiscip CardioVasc Thorac Surg. 2026;41:ivag171. 10.1093/icvts/ivag171 PMC13344852 42406429

[5] Modi P , Hassan A, Teng CJ, et al “How many cardiac surgeons does it take to write a research article?”: seventy years of authorship proliferation and internationalization in the cardiothoracic surgical literature. J Thorac Cardiovasc Surg. 2008;136:4-6. 18603044 10.1016/j.jtcvs.2007.12.057

[6] https://retractiondatabase.org.

[7] Meho LI. Gaming the metrics: bibliometric anomalies in global university rankings and the research integrity risk index (RI^2^). Scientometrics. 2025;130:6683-6726.

[8] Lakhani DA , Radmard M, Yousem DM. Promotion from Associate Professor to Full Professor should not be monolithic: a national bibliometric study by radiology subspecialty. Acad Radiol. 2026 Jun 27:S1076-6332(26)00416-2 10.1016/j.acra.2026.06.004 42362405

